# Development of a deep learning algorithm for myopic maculopathy classification based on OCT images using transfer learning

**DOI:** 10.3389/fpubh.2022.1005700

**Published:** 2022-09-21

**Authors:** Xiaoying He, Peifang Ren, Li Lu, Xuyuan Tang, Jun Wang, Zixuan Yang, Wei Han

**Affiliations:** ^1^Department of Ophthalmology, Eye Center of the Second Affiliated Hospital, School of Medicine, Zhejiang University, Hangzhou, Zhejiang, China; ^2^Department of Ophthalmology, The First Affiliated Hospital, School of Medicine, Zhejiang University, Hangzhou, Zhejiang, China; ^3^Department of Ophthalmology, The First Affiliated Hospital of University of Science and Technology of China, Hefei, Anhui, China

**Keywords:** artificial intelligence, deep learning, pathologic myopia, myopia maculopathy, ATN classification system, optical coherence tomography

## Abstract

**Purpose:**

To apply deep learning (DL) techniques to develop an automatic intelligent classification system identifying the specific types of myopic maculopathy (MM) based on macular optical coherence tomography (OCT) images using transfer learning (TL).

**Method:**

In this retrospective study, a total of 3,945 macular OCT images from 2,866 myopic patients were recruited from the ophthalmic outpatients of three hospitals. After culling out 545 images with poor quality, a dataset containing 3,400 macular OCT images was manually classified according to the ATN system, containing four types of MM with high OCT diagnostic values. Two DL classification algorithms were trained to identify the targeted lesion categories: Algorithm A was trained from scratch, and algorithm B using the TL approach initiated from the classification algorithm developed in our previous study. After comparing the training process, the algorithm with better performance was tested and validated. The performance of the classification algorithm in the test and validation sets was evaluated using metrics including sensitivity, specificity, accuracy, quadratic-weighted kappa score, and the area under the receiver operating characteristic curve (AUC). Moreover, the human-machine comparison was conducted. To better evaluate the algorithm and clarify the optimization direction, the dimensionality reduction analysis and heat map analysis were also used to visually analyze the algorithm.

**Results:**

Algorithm B showed better performance in the training process. In the test set, the algorithm B achieved relatively robust performance with macro AUC, accuracy, and quadratic-weighted kappa of 0.986, 96.04% (95% CI: 0.951, 0.969), and 0.940 (95% CI: 0.909–0.971), respectively. In the external validation set, the performance of algorithm B was slightly inferior to that in the test set. In human-machine comparison test, the algorithm indicators were inferior to the retinal specialists but were the same as the ordinary ophthalmologists. In addition, dimensionality reduction visualization and heatmap visualization analysis showed excellent performance of the algorithm.

**Conclusion:**

Our macular OCT image classification algorithm developed using the TL approach exhibited excellent performance. The automatic diagnosis system for macular OCT images of MM based on DL showed potential application prospects.

## Introduction

Myopic maculopathy (MM) is the main cause for irreversible visual impairment in pathologic myopia (PM) (1). Common signs of MM include chorioretinal atrophy, lacquer cracks, macular hemorrhage, myopic foveoschisis (MF), myopic choroidal neovascularization (mCNV), etc. (2). Among these, macular atrophy, mCNV, and macular hole show great association with the deterioration of visual acuity. Although visual symptoms of MF are often minimal or gradual, it has been demonstrated that MF preceded formation of a macular hole that could eventually lead to retinal detachment. Moreover, the previous literature suggested that pathy chorioretinal atrophy and lacquer cracks are preceding risk factors for the development of mCNV (3).

Myopic maculopathy is a complex disease which cannot be fully described by the current classification systems. The Meta-Analysis of Pathologic Myopia (META-PM) classification, based on the widely used color fundus photography, can only reflect the limited information of main fundus structures, such as morphology and color (4). Although color fundus photography is effective for large-scale screening and identification for various choroidal retinal atrophic lesions, obvious neovascularization, and fundus hemorrhage, it may be insufficient for the minimal, occult, or inner retinal lesion identification. Therefore, more sophisticated examination techniques with comprehensive classification system are desirable in MM research. Optical coherence tomography (OCT), a non-invasive and efficient approach to obtaining high-resolution retinal tomographic images, has been widely used to evaluate various retinal diseases, especially the macular abnormalities in recent years (5).

Artificial intelligence (AI) and DL techniques were firstly applied in ophthalmic fundus photography and have shown good clinical application potential. However, OCT can provide more detailed information of retinal structure, which has great value in the diagnosis of fundus diseases. Recently, with the popularization of OCT, multiple intelligent algorithms for OCT image identification have been developed. Similar to the case in fundus photography, developing the AI-assisted automatic screening and diagnosis system based on OCT images is also of great significance in ophthalmic clinical practice, especially for large scale screening task or community eye care service. Fang et al. developed a novel framework combining convolutional neural networks (CNN) and graph search methods (termed CNN-GS) for automatic segmentation of nine-layer boundaries on retinal OCT images (6). Lee et al. reported satisfying results in automatic segmentation of macular edema OCT images and automatic classification of age-related macular degeneration OCT images using the DL technique (7, 8). The Devalla research team developed a dilated-residual U-Net deep learning network (DRUNET), which can capture both the local and contextual information to segment the individual neural and connective tissues of the optic nerve head tissues in OCT images (9). Perdomo et al. developed a new DL algorithm OCT-NET for diabetic macular edema (10).

Nevertheless, the AI application in myopia is still relatively few. Li et al. trained four independent CNN models to identify retinal holes, macular holes, retinal detachment, and mCNV using macular OCT images of 1,048 highly myopic eyes (11). Using macular OCT images of high myopia, Wei et al. developed a model applying five different deep learning (DL) architectures that can predict the BCVA after cataract surgery and achieved good results (12). Shen et al. developed a DL intelligent system to detect and classify complications of high myopia retinopathy based on OCT images, including macular choroid thinning, macular Bruch membrane loss, subretinal hyperreflective material, myopic tractional maculopathy (MTM), and dome-shaped macula (13). Further development of the comprehensive and intensive AI-assisted automatic identification systems are desired to facilitate the clinical diagnosis and management of MM.

The MM classification and grading system, namely the ATN system, which combines the information of color fundus photographs and OCT images, was proposed by an international team of myopia experts (14). In the ATN classification system, MM lesions are categorized as myopic atrophy maculopathy (MAM), MTM, and myopic neovascular maculopathy (MNM). Among these, the detailed grading of MAM basically refers to the META-PM system, and color fundus photography alone is sufficient in diagnosis and detailed grading. As for diagnosis and detailed grading of MTM and MNM, OCT, fundus angiography (FA), and other examinations have unique advantages. In particular, all grades of macular retinoschisis, macular hole, macular hole with retinal detachment, and active/inactive myopic choroid neovascularization (mCNV) are easy to be distinguished with OCT images.

In our previous work, DL showed comparable ability of ophthalmic fundus image classification and recognition when compared with the ordinary ophthalmologists, and even approached the level of retinal specialists (15). Meanwhile, transfer learning (TL) has been widely used in the development of medical DL algorithms, which means the infrastructure used is pre-trained in the huge ImageNet database, and then optimized with medical image data. In this study, we aimed to develop an automatic classification system using DL technology to identify the different types of MTM and MNM based on the macular OCT images. Combined with the automatic classification of MAM in our previous research, the full coverage of the intelligent automatic classification and diagnosis of MM using the ATN system was preliminary materialized. In addition, we also developed a new OCT classification algorithm using the TL approach based on the excellent classification algorithms (Algorithm I and Algorithm II) which was developed in our previous study (15).

## Methods

### Data collection

In this study, the use of OCT images was approved by the Ethics Committee of the First Affiliated Hospital, School of Medicine, Zhejiang University and adhered to the tenets of the Declaration of Helsinki. Because the study was a retrospective review and analysis of fully anonymized OCT images, the medical ethics committee declared it exempt from informed consent.

We collected 3,945 OCT images of 2,866 myopia patients (Table 1) from the eye center of the First Affiliated Hospital of School of Medicine, Zhejiang University; the First Affiliated Hospital of University of Science and Technology of China; and the First Affiliated Hospital of Soochow University between Jan 2020 and Jan 2021. Since February 2021, the manual annotation and algorithm training of OCT images have been carried out. All images were captured on two different SD-OCT (Heidelberg Spectralis HRA + OCT and Rtvue XR). All three eye centers used a similar imaging protocol, a horizontal or vertical single-line scan (10 mm) through the fovea. The pupil dilation was decided by the examiners depending on the patient's ocular condition. Patient information attached to images was anonymized before inclusion in the study.

**Table 1:** Summary of the total dataset and external validation dataset.

	**Number of images with labels**	**Number of participants**	**Mean age (years)**	**Sex (% female)**	**Spherical equivalent (diopters)**
**Total dataset**					
Normal macular	1,025	686	40.22 ± 7.78 (18 to 67)	52.2	1.13 ± 0.83 (−6.5 to −0.5)
Macular schisis	506	308	54.71 ± 12.52 (28 to 69)	57.6	−16.78 ± 4.01 (−22 to −8)
Full-thickness macular hole	556	387	55.48 ± 10.32 (29 to 64)	63.1	−15.32 ± 6.84 (−21.5 to −8.5)
MH with retinal detachment	230	213	58.19 ± 10.11 (28 to 69)	58.9	−16.45 ± 5.79 (−22.5 to −9.0)
mCNV	570	470	52.15 ± 12.12 (29 to 78)	57.6	−14.87 ± 3.23 (−21 to −7)
Others	513	402	46.71 ± 10.78 (29 to 70)	51.9	−1.07 ± 0.63 (−4.5 to −0.5)
**External validation dataset**					
Normal macular	67	40	38.05 ± 14.47 (26 to 75)	53.1	−1.34 ± 0.81 (−5.5 to −0.5)
Macular schisis	187	141	57.41 ± 13.12 (30 to 71)	59.4	−17.98 ± 4.52 (−24 to −8)
Full-thickness macular hole	219	187	58.19 ± 12.37 (24 to 65)	57.4	−16.35 ± 7.16 (−23.5 to −6.5)
MH with retinal detachment	148	109	61.02 ± 11.71 (27 to 71)	60.1	−17.18 ± 7.04 (−23.5 to −8.5)
mCNV	159	135	54.724 ± 13.52 (27 to 81)	56.3	−15.37 ± 3.04 (−20 to −7)
Others	220	201	49.24 ± 11.38 (28 to 67)	53.2	−2.24 ± 0.84 (−5 to −0.5)

mCNV, myopic choroid neovascularization; MH, macular hole.

The inclusion/exclusion criteria of the images were as follows: Images obtained from eyes with refractive error (spherical equivalent ≤ −0.50 D). Images of any MM must be from highly myopic eyes (spherical equivalence ≤ −6.00 D). Images obtained from eyes with MM accompanied by other macular lesions were excluded. As the accurate preoperative refractive error of patients had refractive surgery history could not be obtained directly, the patients with previous refractive surgery history were not involved. At the same time, images with poor quality caused by cataracts, vitreous opacities, eye movement, or other reasons were excluded to ensure readability. Finally, a total of 545 ungradable images were excluded. A total dataset containing 3,400 macular OCT images was established and assigned to the expert teams for further annotation.

### Diagnostic reference standard and manual annotation

**Figure 1:**
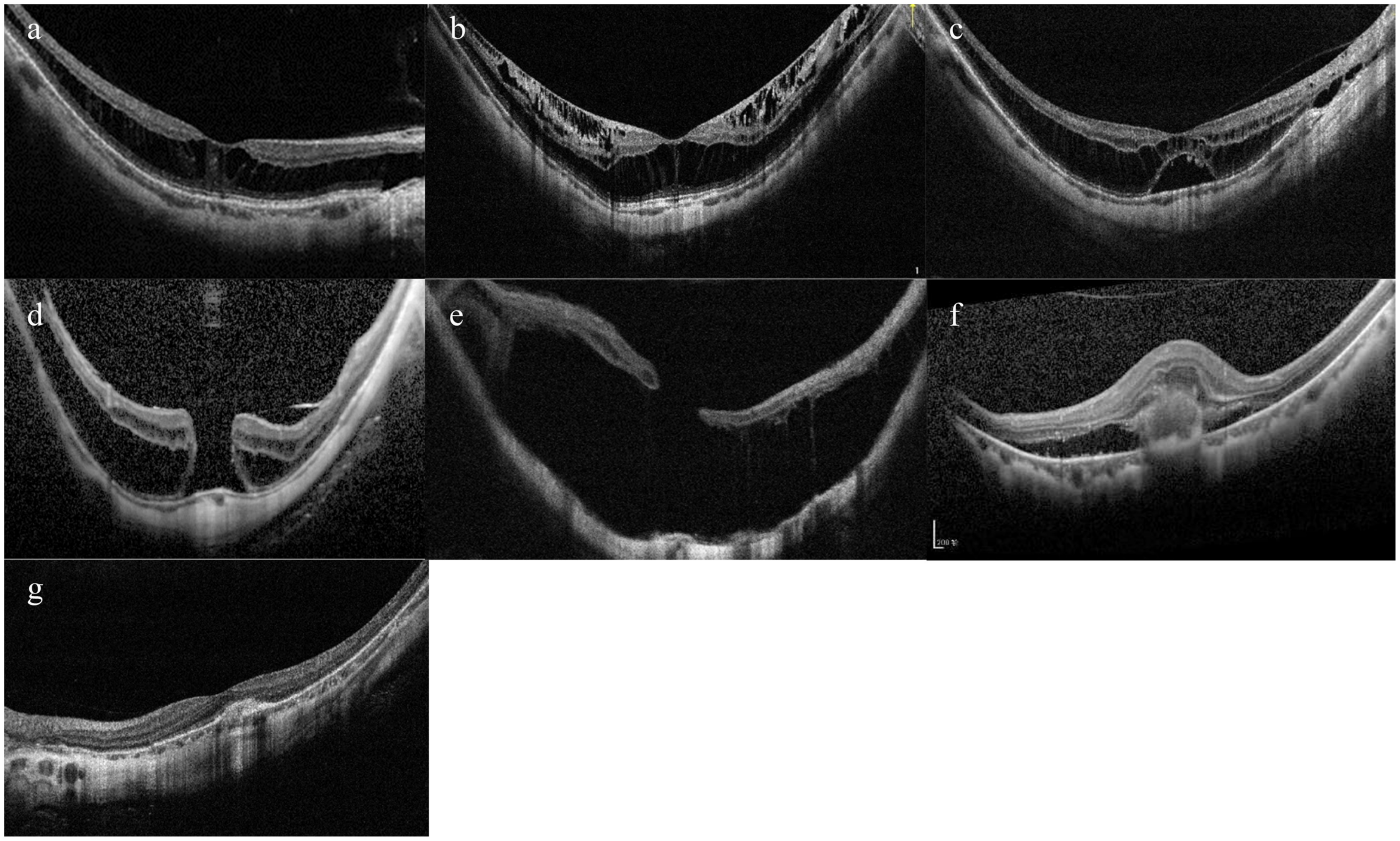
Typical OCT images of tractional component and neovascular component. **(A)** Inner or outer foveoschisis (T1). **(B)** Inner + outer foveoschisis (T2). **(C)** Foveal detachment (T3). **(D)** Full-thickness Macular hole (MH) (T4). **(E)** MH + Retinal detachment (T5). **(F)** Active CNV (N2a). **(G)** Scar/Fuch's spot (N2s).

According to the ATN classification and grading system, MM was divided into three types—MAM, MTM, and MNM (14). Specifically, MAM is subdivided into A0–A4, which basically corresponds to C0–C4 in the META-PM classification (4). Myopic tractional maculopathy was subdivided into T0—no macular schisis, T1—inner or outer foveoschisis, T2—inner + outer foveoschisis, T3—foveal detachment, T4—full-thickness macular hole (MH), T5—MH with retinal detachment. Myopic neovascular maculopathy was subdivided into N0—no mCNV, N1—macular lacquer cracks, N2a—active CNV, N2s—scar/Fuch's spot. Typical OCT images of MTM and MNM were shown in Figure 1.

**Figure 2:**
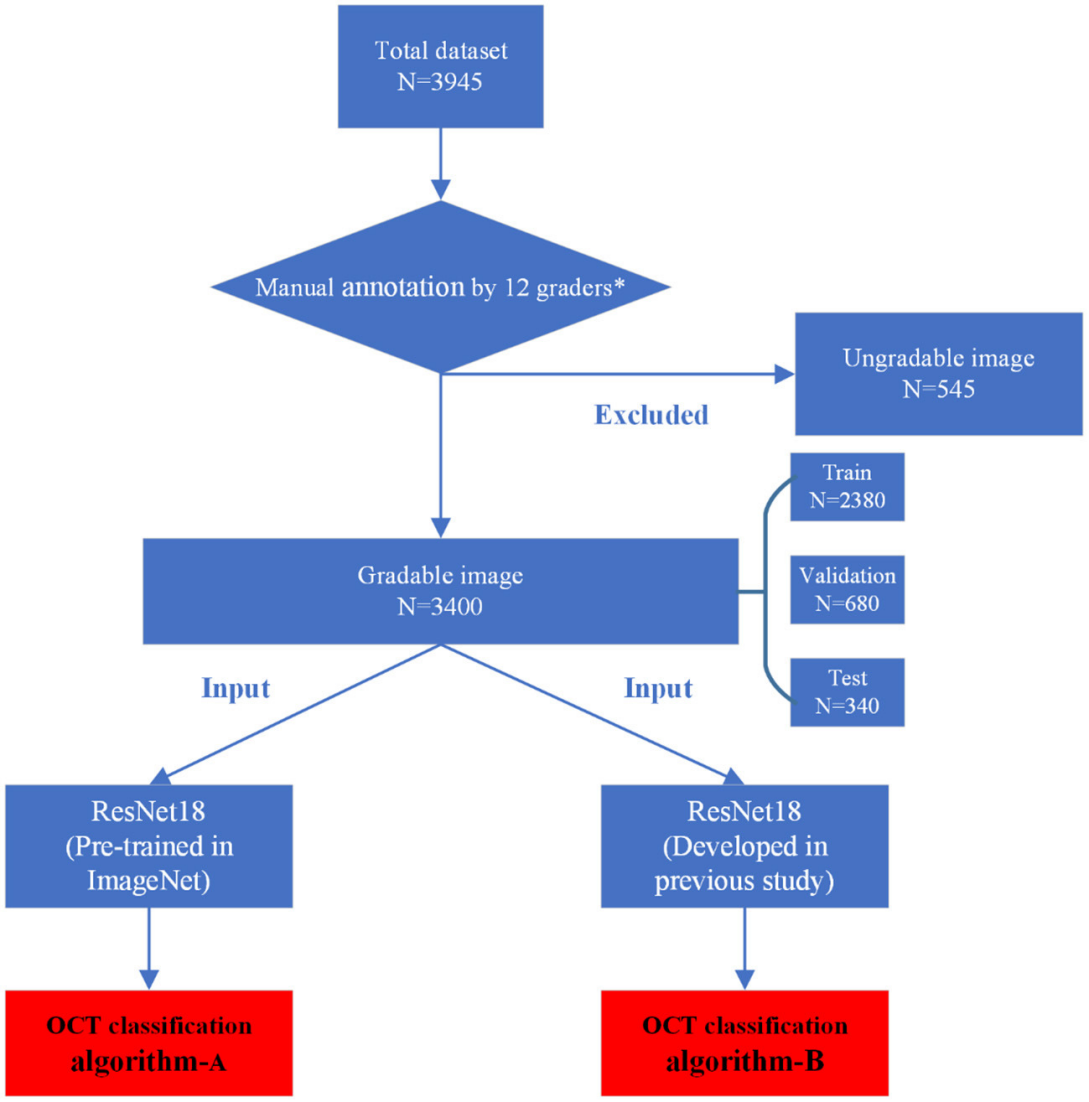
Workflow diagram showing the overview of the development process.

The manual annotation protocol was in accordance with our previous study (16). The 12 ophthalmologists were required to learn the definition and test the intra- and inter-rater reliability before starting the annotation process. After achieving a kappa value ≥0.81 (almost perfect), the 12 ophthalmologists served as the graders (17). They were randomly divided into three teams, with each consisting of one retinal specialist (with more than 10 years of experience) and three general ophthalmologists (with more than 5 years of experience). Graders in the same team evaluated the same set of images. Each grader was blinded to the annotation results made by the others and the independent decisions on the OCT images were made. The results recognized unanimously by the three graders in the same team were taken as the ground truth. Results that differed among the general ophthalmologists in the same team were arbitrated by the retinal specialist for a final decision of annotation. The workflow of manual annotation was illustrated in Figure 2.

In this study, in view of the feasibility of image data acquisition, accuracy of manual diagnosis, and clinical significance of the lesions involved, the T1, T2, and T3 were combined as macular schisis; and the active mCNV (N2a) and scar/Fuchs spots (N2s) were combined as mCNV. The single modality of OCT images is not sensitive in the diagnosis of macular LCs, which relies on FA or multimodal diagnosis of near-infrared reflectance (NIR) images combined with OCT images (18), and is therefore not addressed in this study. If multiple myopic traction maculopathy were found to be present on the same OCT image, only the highest level was retained. After manual annotation, no mCNV images were found to be combined with myopic traction maculopathy, so each image carried only one lesion label. In general, 3,400 OCT images were manually classified as normal macular, macular schisis, full-thickness macular hole, MH with retinal detachment, mCNV, and the others.

### Development of the deep learning algorithms

After being processed by manual annotation, the total dataset was randomly divided into the training dataset, validation dataset, and test dataset, accounting for 70%, 20%, and 10%, respectively. Each image was allowed to exist in one dataset. All the raw OCT images were pre-processed by cropping and resizing to meet the requirement of input image format with a resolution of 512 ^*^ 512 pixels. The images in the training and validation datasets were pre-processed by the steps of gray-scale transformations, geometric variation, and image enhancement, in order to eliminate the irrelevant information and recover the useful or true information in images. The features of input images were then analyzed and extracted by the DL algorithm. The results of multiclass classification were given. The training platform was implemented with the PyTorch framework, and all of the DLSs were trained in parallel on four NVIDIA 2080 Ti graphics processing units (16).

**Figure 3:**
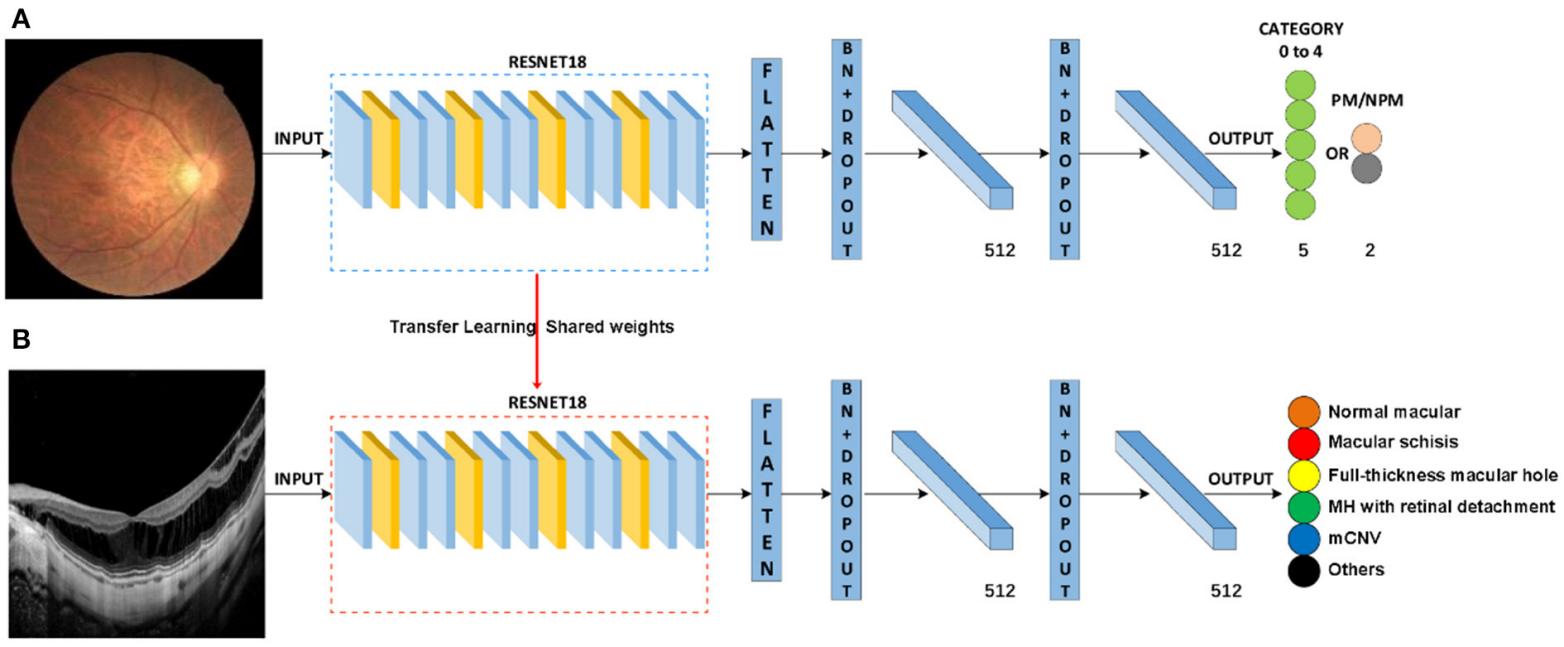
Schematic diagram of algorithm architecture. **(A)** Architecture of classification algorithms in our previous study. **(B)** OCT image classification algorithm is constructed by the transfer learning method.

In this study, a total of two DL classification algorithms were trained to identify the targeted lesion categories, and the workflow was illustrated in Figure 2. The OCT classification algorithm A, using the same deep residual network model architecture reported in our previous work (15), was pre-trained by ImageNet with no initialization parameters, and then the labeled OCT images were added to finalize the training. The OCT classification algorithm B was based on the classification algorithm developed in our previous study (15), using a multi-source cross-domain TL approach, sharing the prior distribution of model parameters, learning the feature representation of the OCT images, and further training to optimize the initialization parameters of the model. Details of the relevant architecture were shown in Figure 3.

### Retrospective external validation and expert-machine comparison

To further evaluate our algorithm, 1,000 OCT images from 813 myopic patients were recruited retrospectively according to the same criteria at the Second Affiliated Hospital of School of Medicine, Zhejiang University as an external validation dataset (Table 1). The model of OCT was the same as the one used in the training dataset. The protocol for the human-machine comparison was consistent with our previous study (15).

### Visualization analysis: Dimensionality reduction and heat map

We used a t-distributed Stochastic Neighbor Embedding (t-SNE) method to reduce high-dimensional DL data features to two-dimensions and visualize them to observe the feature subspace aggregation capability of the algorithm. The t-SNE converts similarities between data points and minimizes the Kullback–Leibler divergence of the joint probabilities between the low-dimensional embedding and the high-dimensional data (19). The heat map analysis approach was consistent with our previous study (16).

### Statistics and reproducibility

The accuracy and cross-entropy loss curves of the two algorithms during training were also recorded and compared. The better one was selected to be further tested in the test dataset and external validation dataset. The performance of the algorithm in classifying lesions was evaluated using metrics including sensitivity, specificity, and the area under the receiver operating characteristic curve (AUC). The area under the macro average of ROC (macro-AUC) for each class in a one-vs.-all manner and quadratic-weighted kappa score was calculated. The accuracy was recorded as the number of images judged correctly in all classifications divided by the total number of images participating in the test. The confusion matrices were also demonstrated. Additionally, the Clopper-Pearson method was used to calculate the 95% CI. Statistical data were analyzed using Sigma Plot 14.0 and Python 3.7.3. The detailed calculation formulas are as follows:


$$\begin{array}{ccc} sensitivity & = & \frac{TP}{TP+FN}\\ specificity & = & \frac{TN}{FP+TN}\\ accuracy & = & \frac{TP+TN}{TP+FP+TN+FN} \end{array}$$

*TP*: true positive, *FN*: false negative, *FP*: false positive, *TN*: true negative.

## Results

Our total dataset was filtered from 3,945 OCT scans to 3,400 gradable images for lesions classification. The workflow is shown in Figure 2. Approximately 10% of the images were subjected to a final adjudication by retinal experts. The characteristics and summary of the dataset are shown in Table 1.

### Comparison of the training process of the two algorithms

**Figure 4:**
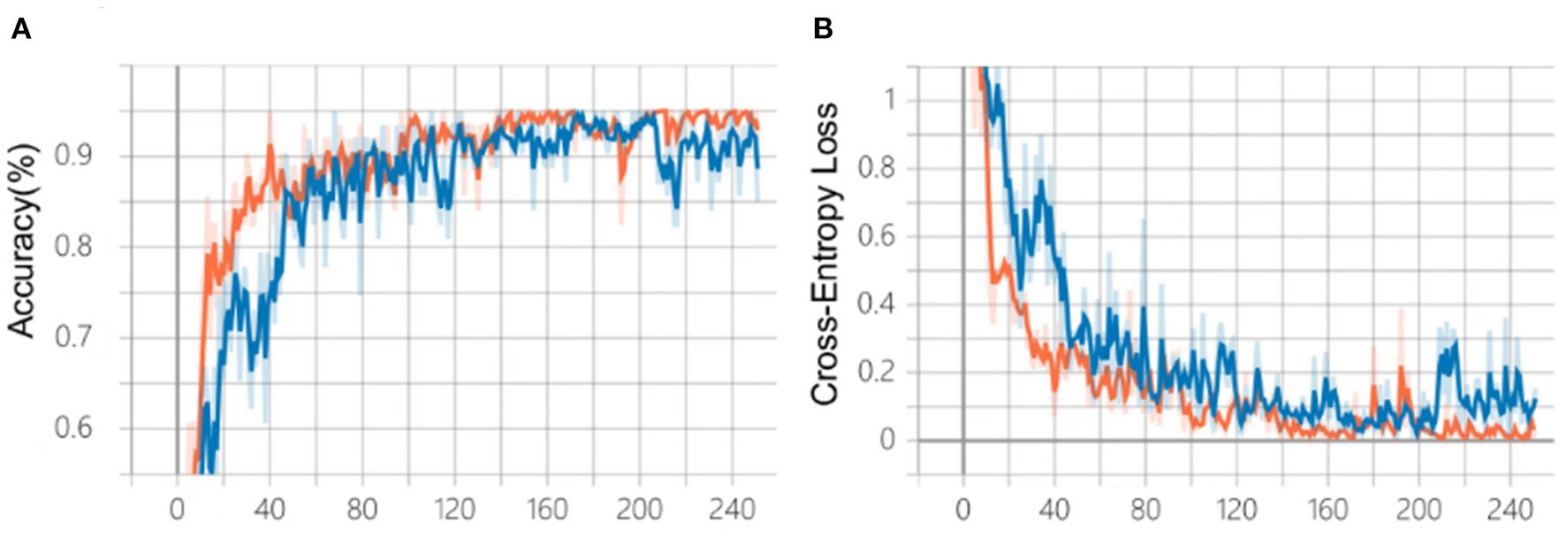
Comparison of the training process of the two algorithms. **(A)** Changes in accuracy during training. **(B)** Changes of cross-entropy in training. Blue line: algorithm A; Orange line: algorithm B.

After 240 epochs, the training of the two algorithms ceased as the accuracy and cross-entropy loss did not improve further (Figures 4A,B). After comparison based on the fundus photo classification algorithm developed in our previous study, algorithm B, which was trained based on the multi-source cross-domain TL approach, was found to achieve faster accuracy improvement and cross-entropy loss reduction. At the end of the training, the accuracy of algorithm B was about 95%, which was higher than that of algorithm A.

### Performance of the algorithm B in the test dataset

**Figure 5:**
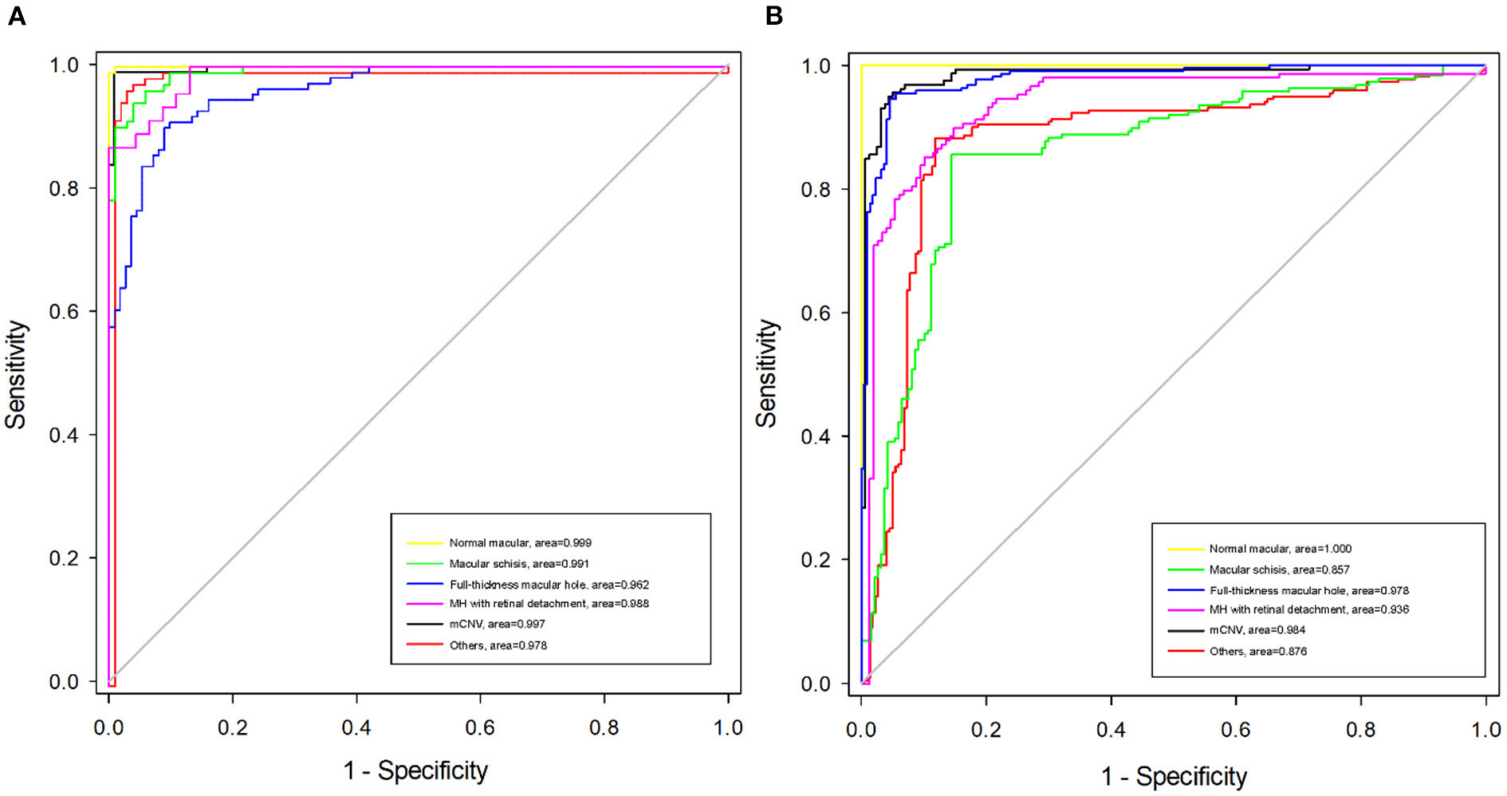
Receiver operating characteristic (ROC) curves of algorithm B in test dataset and external validation dataset. **(A)** The ROC curve of algorithm B in the test dataset. **(B)** The ROC curve of algorithm B in the external validation dataset.

**Figure 6:**
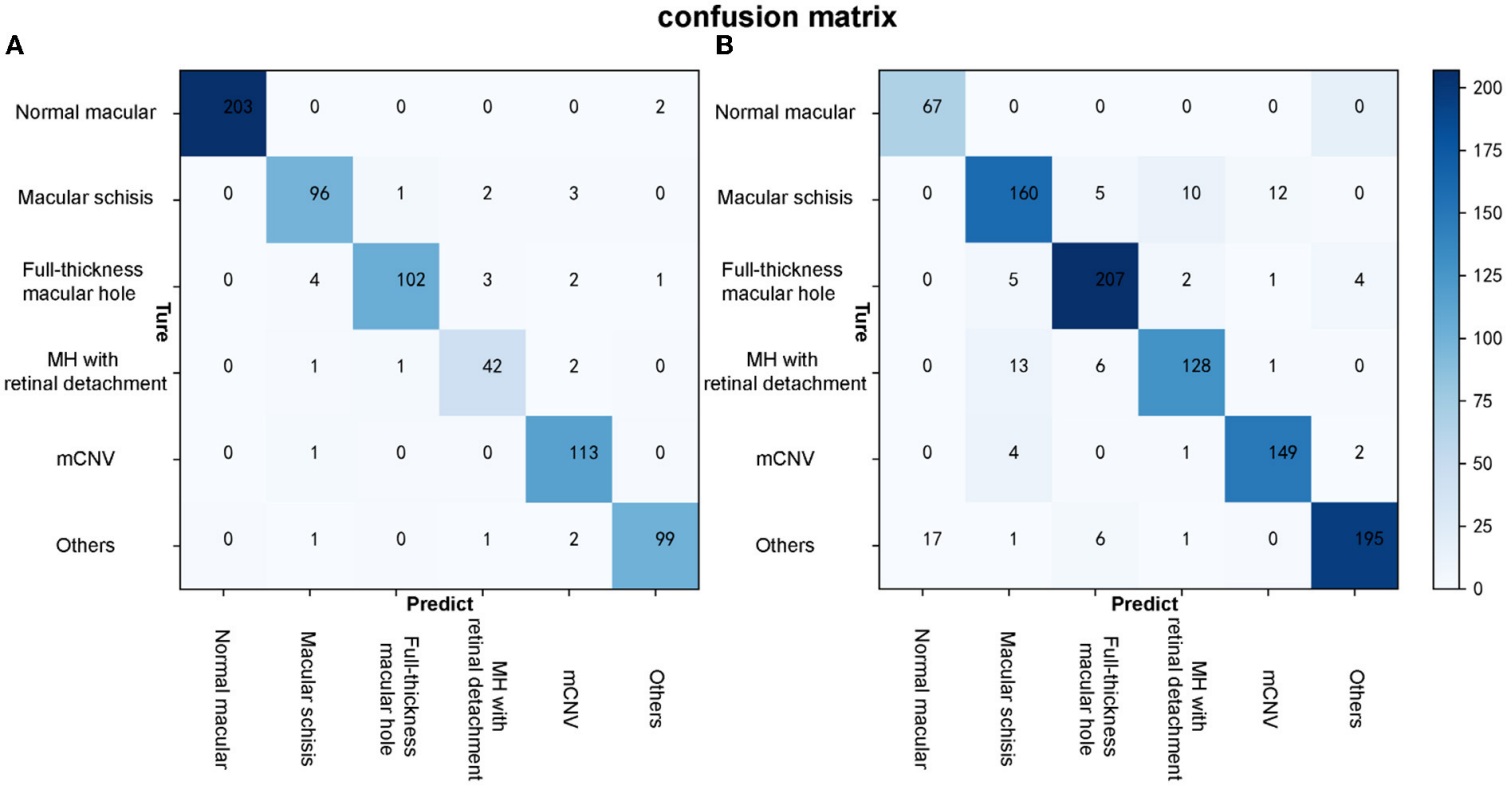
The confusion matrixes of algorithm B in both test and external validation datasets. **(A)** The confusion matrix of algorithm B in the test dataset. **(B)** The confusion matrix of algorithm B in the external validation dataset.

Algorithm B achieved good performance in the test dataset. Specifically, algorithm B achieved a macro AUC = 0.986 (95% CI: 0.979, 0.993), accuracy = 96.04% (95% CI: 0.951, 0.969), and a quadratic weighted kappa value = 0.940 (95% CI: 0.909, 0.971) in the classification task (Table 2, Figure 5A). For specific lesion identification, the AUC of algorithm B for macular schisis was 0.991 (95% CI: 0.998, 1.000), sensitivity was 94.12% (95% CI: 0.924, 0.959), and specificity was 98.79% (95% CI: 0.980, 0.996); the AUC for full-thickness MH was 0.962 (95% CI : 0.953, 0.971), sensitivity was 91.07% (95% CI: 0.889, 0.932), specificity was 99.65% (95% CI: 0.992, 1.000); AUC for MH with Retinal detachment was 0.988 (95% CI: 0.983, 0.993), sensitivity was 91.30% (95% CI: 0.892, 0.934) with a specificity of 99.06% (95% CI: 0.983, 0.998); the AUC for mCNV was 0.997 (95% CI: 0.994, 0.999) with a sensitivity of 99.12% (95% CI: 0.984, 0.998) and a specificity of 98.42% (95% CI: 0.975, 0.994); for others the AUC was 0.978 (95% CI: 0.971, 0.985), sensitivity was 96.12% (95% CI: 0.947, 0.976), and specificity was 99.48% (95% CI: 0.989, 1.000) (Table 2). The confusion matrix for algorithm B in the test dataset is shown in Figure 6A.

**Table 2:** The performance of the algorithm B in the test dataset.

	**Macro-AUC**	**Accuracy**	**Quadratic-weighted kappa**
	**(95% CI)**	**(95% CI)**	**(95% CI)**
**Algorithm B**	0.986	0.960	0.940
	(0.979, 0.993)	(0.951, 0.969)	(0.909–0.971)
**Classification**	**AUC** **(95% CI)**	**Sensitivity** **(95% CI)**	**Specificity** **(95% CI)**
Macular schisis	0.991 (0.998, 1.000)	94.12% (0.924, 0.959)	98.79% (0.980, 0.996)
Full-thickness macular hole	0.962 (0.953, 0.971)	91.07% (0.889, 0.932)	99.65% (0.992, 1.000)
MH with retinal detachment	0.988 (0.983, 0.993)	91.30% (0.892, 0.934)	99.06% (0.983, 0.998)
mCNV	0.997 (0.994, 0.999)	99.12% (0.984, 0.998)	98.42% (0.975, 0.994)
Others	0.978 (0.971, 0.985)	96.12% (0.947, 0.976)	99.48% (0.989, 1.000)

AUC, area under the receiver operating characteristic curve; CI, confidence interval; mCNV, myopic choroid neovascularization; MH, macular hole.

### Performance of algorithm B in the external validation dataset and expert-machine comparison

**Figure 7:**
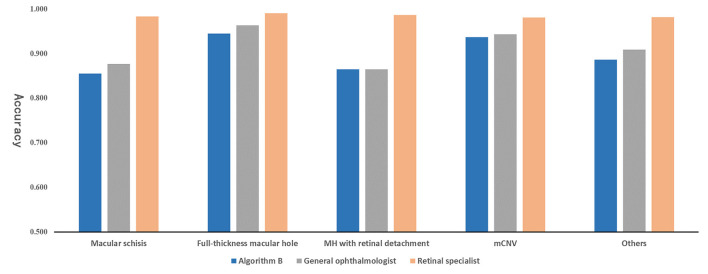
The comparison between algorithm B and experts on accuracy in external validation.

Algorithm B performed remarkably well in the test dataset. To further evaluate the performance and generalizability of the algorithm B, we retrospectively collected 1,000 OCT images as the external validation dataset. Algorithm B performed slightly worse in the external validation dataset than in the test dataset (Figures 5B, 6B). This result was consistent with the previous research (8). In detail, algorithm B has regressed in its performance in the identification of macular schisis, MH with retinal detachment. In the expert-machine comparison, there was indeed some gap between the accuracy of algorithm B and that of the retinal specialist. However, the performance has been very close to the general ophthalmologist. The difference in the judgment of specific individual lesions was within 3% in terms of accuracy, sensitivity, and specificity (Table 3, Figure 7).

**Table 3:** Comparison of the performance of the algorithm B and experts in external validation dataset.

	**Macro-AUC** **(95% CI)**			**Accuracy** **(95% CI)**		**Quadratic-weighted kappa** **(95% CI)**	
**Algorithm B**	0.938 (0.923, 0.953)			0.906 (0.888, 0.924)		0.897 (0.871, 0.922)	
**General ophthalmologist**	NA			0.915 (0.898, 0.932)		0.928 (0.907, 0.950)	
**Retinal specialist**	NA			0.958 (0.978, 0.993)		0.991 (0.985, 0.997)	
	**Algorithm B**			**General ophthalmologist**		**Retinal specialist**	
**Classification**	**AUC** **(95% CI)**	**Sensitivity** **(95% CI)**	**Specificity** **(95% CI)**	**Sensitivity** **(95% CI)**	**Specificity** **(95% CI)**	**Sensitivity** **(95% CI)**	**Specificity** **(95% CI)**
Macular schisis	0.857 (0.839, 0.913)	0.856 (0.834, 0.877)	0.972 (0.961, 0.982)	0.877 (0.857, 0.897)	0.988 (0.981, 0.995)	0.984 (0.976, 0.992)	0.995 (0.991, 0.999)
Full-thickness macular hole	0.978 (0.966, 0.990)	0.945 (0.931, 0.959)	0.974 (0.965, 0.984)	0.964 (0.952, 0.975)	0.978 (0.969, 0.987)	0.991 (0.985, 0.997)	0.999 (0.997, 1.000)
MH with retinal detachment	0.936 (0.905, 0.967)	0.865 (0.844, 0.886)	0.984 (0.976, 0.991)	0.865 (0.844, 0.886)	0.987 (0.980, 0.994)	0.987 (0.979, 0.994)	0.999 (0.997, 1.000)
mCNV	0.984 (0.970, 0.997)	0.937 (0.922, 0.952)	0.983 (0.975, 0.991)	0.943 (0.929, 0.958)	0.987 (0.979, 0.994)	0.981 (0.973, 0.989)	0.996 (0.993, 1.000)
Others	0.876 (0.839, 0.913)	0.890 (0.871, 0.910)	0.992 (0.987, 0.998)	0.909 (0.891, 0.927)	0.958 (0.945, 0.970)	0.982 (0.974, 0.990)	0.994 (0.989, 0.999)

AUC, area under the receiver operating characteristic curve; CI, confidence interval; mCNV, myopic choroid neovascularization; MH, macular hole; NA, not available.

### Visual analysis of algorithm B in the test dataset

**Figure 8:**
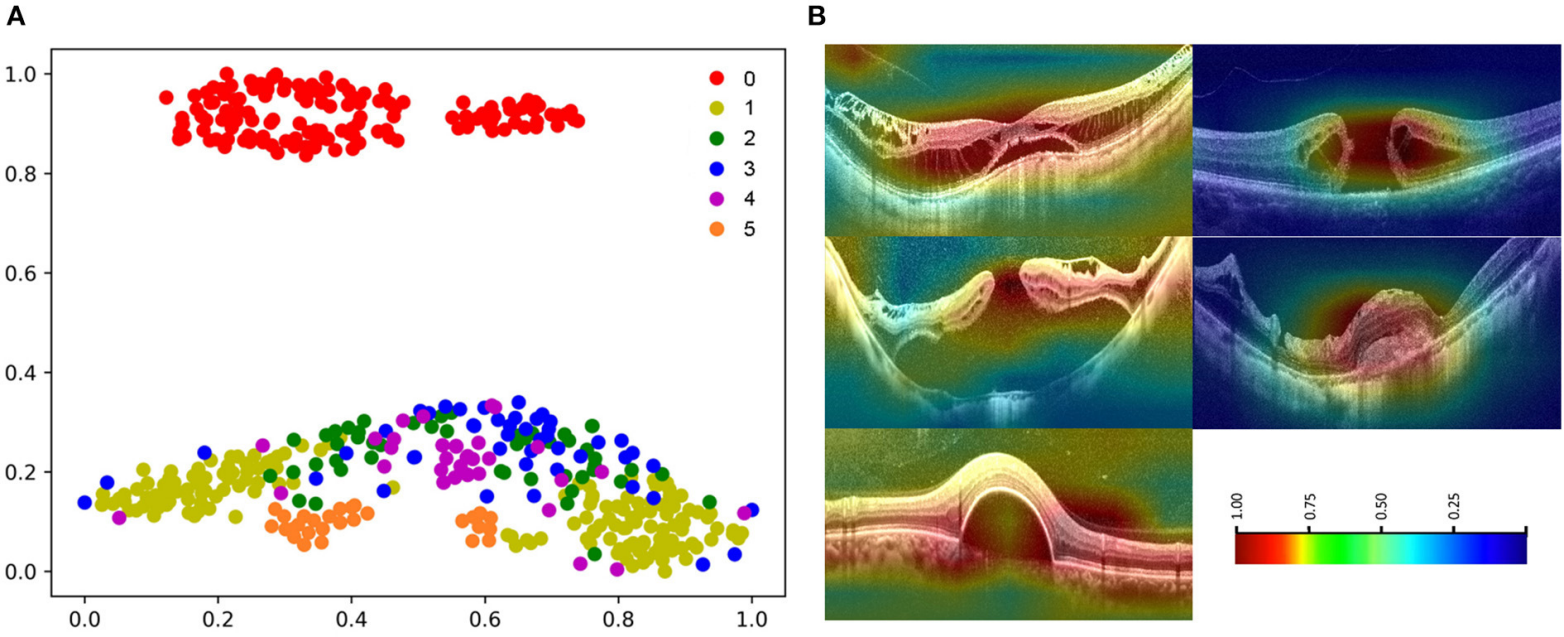
Visualization of algorithm B. **(A)** The t-SNE plot of algorithm 0: normal macular, 1: macular schisis, 2: full-thickness macular hole, 3: MH with Retinal detachment, 4: mCNV, 5: others (PED). **(B)** Heatmap generated from deep features overlaid on the original images. The typical lesions were observed in the hot regions.

The high-dimensional features of the classification algorithm were visualized in Figure 8A after the dimensionality reduction analysis of t-SNE. In the t-SNE plot, clustering occurred for the same label and significant feature space differences existed between different labels, indicating the good classification performance of the algorithm. Figure 8B showed the results of visual heat map analysis of algorithm B. The OCT images of different lesions were processed by the visualization layer and a heat map was attached to the original image. The algorithm highlighted the areas on the original image that were most critical to its classification judgment with hot color, and the retinal specialist then assessed the consistency of the hot zones with the actual lesion areas in the OCT image. The main lesion areas including macular schisis, full-thickness macular hole, MH with retinal detachment, mCNV, and others (PED) were found to be within the hot zone, and the accuracy of their extent was confirmed by the retinal specialist.

## Discussion

The present study is an extension of our previous work and demonstrates the potential of DL technique in processing various modalities of ophthalmic imaging data. In this work, two DL classification algorithms, algorithm A and B, were trained to automatically identify six different types of macular OCT images. Although the same algorithm infrastructure was applied for the two algorithms, faster improvement of accuracy and cross-entropy loss reduction were achieved with algorithm B, which was developed using the multi-source cross-domain TL approach based on the fundus photo classification algorithm as described in our previous study (15). At this stage, despite the certain gap between algorithm B and retinal experts, the performance indicators of our algorithm basically reached the level of general ophthalmologists (Figure 7).

In our algorithm development, the ATN classification system was adopted as the gold standard, which provides a unified standard for the image diagnostic standards and relevant management for the MM. Four kinds of macular lesions sensitive to OCT examination in MTM and MNM were included for further research. Considering the actual clinical setting, normal macular and other macular degeneration categories were also included. In fact, MM grades C0–C4 in the META-PM classification system overlap with MAM grades A0–A4 in the ATN classification system, while the “Plus” lesions in the META-PM classification system are similar to MNM in the ATN classification system. Therefore, our present study achieved a comprehensive intervention of DL techniques in the main category of MM based on the ATN classification system. However, in the external validation dataset containing 1,000 macular OCT images constructed to further test the effectiveness and generalizability of our algorithm, the slight degradation of the algorithm performance was observed. This is within the expectation and can be ascribed to the discrepancy between the test data sources. Nevertheless, the results in the external dataset were basically acceptable (Figure 5, Table 3).

In our work, the TL approach played a critical role in the training and testing process of the OCT classification algorithm. Briefly, the TL approach is the application of knowledge or feature distribution learned in a certain domain or task to a different but related new domain or task. The purpose is to transfer the network parameter distribution of the labeled data or knowledge structure learned by the neural network model from related fields and to initialize the neural network to complete or improve the learning effect of the target field or task (20). At present, the TL approach has been widely used in the development of algorithms for medical image analysis. The most common process of TL approach is as follows: Firstly, pre-train the algorithm model using public large-scale non-medical professional databases (such as ImageNet). Secondly, fine-tune or freeze the convolutional layer of the model (keep the parameters unchanged). Finally, retrain the neural network and the fully connected classification layer with the labeled medical professional data. TL approach can speed up the convergence speed of the network and improve the training accuracy of the network. The effect of TL is based on the number of fine-tuning, the choice of model architecture, the order of fine-tuning, and the choice of pre-training database (21). The pre-training database should be as close as possible to the current task database to avoid unsatisfying results. In this study, we choose the color fundus photography classification algorithm developed in our previous study as the starting point for the development of the OCT image algorithm and shared its model parameters and weights. As expected, algorithm B, transferred from the previous eye image classification algorithm, outperformed algorithm A (pre-trained on ImageNet) developed from scratch (Figure 4).

The visual heatmap analysis was used to show the areas where the algorithm considered a positive contribution to the classification results. The good correspondence between the hot areas and the actual lesions in macular OCT images validated the effectiveness of our OCT classification algorithm from a clinical perspective (Figure 8). Of note, we applied a novel visualization approach termed dimensionality reduction analysis, which comprises the most effective non-linear dimensionality reduction method termed t-SNE. The dimensionality reduction analysis facilitates the understanding and validation of the data or model through visualization. Moreover, the t-SNE plots of images with the same label clustered, while the t-SNE plots of images with different labels showed significant differences, suggesting the good performance of the algorithm (Figure 8).

It should be noted that there are three detailed subdivisions for macular retinoschisis (inner or outer retinoschisis, inner and outer retinoschisis, and macular detachment) and three detailed subdivisions for MNM (LCs, active mCNV, and scars/Fuchs spots) in the ATN classification system. However, our work merged the two sub-level macular OCT images and classified them into two major categories: macular retinoschisis and mCNV. The reason is mainly due to the relatively limited data volume. Certainly, it is better to classify the each subdivision level separately, but a huge amount of data would be required for training each classification so as to obtain a satisfying result. Importantly, our algorithm at this stage aims to serve as a screening tool from clinical application perspective. Therefore, it is acceptable to adopt a broader classification system to achieve better accuracy and efficiency. With the continuous accumulation of subdivision-level image data, the more refined OCT classification algorithm that fully corresponds to the ATN classification system will be expected in our future work.

Despite the high diagnostic value of macular OCT images for MTM and MNM in the ATN classification system, the diagnosis was still made based on a single-modal algorithm after all. Currently, the multiple clinical methods for fundus examination are available, including color fundus photography, OCTA, and FA, etc. Fundus angiography is the gold standard for the diagnosis of mCNV, while OCTA also play important role in clinical practice. The color fundus photography has a good performance in the diagnosis of MAM, but macular OCT has a good discriminative ability for MAM with the measurement of choroidal thickness and scleral thickness (22, 23). Therefore, the algorithms of multi-modal intelligent diagnosis of MMs based on the ATN classification system will definitely be more robust for automatic identification of the lesions and deserves further investigation.

Although the DL algorithm developed in our study achieved satisfactory performance in identification of the six categories of macular OCT images, misclassification might still exist. The potential contributory factors include: (i) The quality of OCT images. Although images with poor quality caused by obvious cataracts, corneal scar, vitreous opacities, eye movement, etc. were excluded to ensure readability, some images with relative low quality might still exist due to the mild opacity of the refractive medium, small pupil diameter, and long eye axis of high myopia, which can be recognized accurately by human experts but might be misclassified using DL algorithm. (ii) The existence of multiple MTM lesions in one image. Although only the highest lesion level was retained, the co-existence of multiple types of MTM in one image might affect the classification to some extent. (iii) The small dataset. Our dataset for training was relatively small. The images with atypical occult lesions with various morphology or position might be misclassified. A larger dataset containing typical and atypical lesions will be desirable in our further work.

In conclusion, according to the ATN classification system, our study developed a DL algorithm using TL approach based on macular OCT images and achieved the automatic intelligent identification of the six categories of macular OCT images (including macular retinoschisis, full-thickness macular hole, macular hole with retinal detachment, mCNV, normal macula, and other macular lesions). The performance of the algorithm developed at this stage was satisfactory for the mission of relevant lesions classification. The present study demonstrates the potential application of the intelligent algorithms in the ophthalmic clinical tasks, especially for the highly myopic fundus lesions identification. Combined with our previous work (15, 16), this work is also a part of our ongoing effort to develop the multiple-modal algorithms for automatic diagnosis of MM based on the multiple fundus examination methods including color fundus picture, OCT, OCTA, and FFA/ICGA, etc.

## Data availability statement

The original contributions presented in the study are included in the article/supplementary material, further inquiries can be directed to the corresponding author/s.

## Ethics statement

The studies involving human participants were reviewed and approved by the Ethics Committee of the First Affiliated Hospital, School of Medicine, Zhejiang University. Written informed consent for participation was not required for this study in accordance with the national legislation and the institutional requirements.

## Author contributions

XH, LL, and WH: design of the study. PR, XT, JW, and ZY: acquisition, analysis, or interpretation of the data. XH, PR, and LL: drafting of the manuscript. LL: statistical analysis. WH: obtaining the fund and supervising the process. All authors revision and approval of the manuscript.

## Funding

This study was supported by grant from the Science and Technology Project of Zhejiang Province (Grant No. 2019C03046).

## Conflict of interest

The authors declare that the research was conducted in the absence of any commercial or financial relationships that could be construed as a potential conflict of interest.

## Publisher's note

All claims expressed in this article are solely those of the authors and do not necessarily represent those of their affiliated organizations, or those of the publisher, the editors and the reviewers. Any product that may be evaluated in this article, or claim that may be made by its manufacturer, is not guaranteed or endorsed by the publisher.
